# Hypoxia in human soft tissue sarcomas: Adverse impact on survival and no association with *p53* mutations

**DOI:** 10.1054/bjoc.2001.1728

**Published:** 2001-04

**Authors:** M Nordsmark, J Alsner, J Keller, O S Nielsen, O M Jensen, M R Horsman, J Overgaard

**Affiliations:** Danish Cancer Society Department of Experimental Clinical Oncology and Departments of; Oncology; Orthopaedic Surgery and; Pathology, Aarhus University Hospital, Denmark

**Keywords:** soft tissue sarcoma, *p53*, hypoxia, polarographic oxygen electrode, tumour oxygenation, predictive assay, survival

## Abstract

Clinical and experimental studies have suggested that tumour hypoxia is associated with poor treatment outcome and that loss of apoptotic potential may play a role in malignant progression of neoplastic cells. The tumour suppressor gene *p53* induces apoptosis under certain conditions and microenvironmental tumour hypoxia may select for mutant tumour cells with diminished apoptotic potential due to lack of p53 function. The aim of this study was to evaluate the prognostic relevance of oxygenation status for treatment outcome and to compare pre-treatment tumour oxygenation measurements were done in 31 of those by PCR using DNA extracted from paraffin-embaedded sections (*n* = 2) or frozen biopsies (*n* = 29). The overall median of the tumour median pO _2_ was 19 mmHg (range 1–58 mmHg). Only 6 tumours had functional p53 mutations and no association was found between mutant p53 and tumour hypoxia. Five out of 6 STS with lower histopathological grade were well-oxygenated whereas high-grade STS were both hypoxic and well-oxygenated. At a median follow-up of 74 months, 16 patients were still alive among 28 available for survival analysis. When stratifying into hypoxic and well-oxygenated tumours patients with the most hypoxic tumours has a statistically poorer disease-specific and overall survival at 5 years. In conclusion hypoxia was an indicator for both a poorer disease specific and overall survival in human STS but hypoxic tumours were not characterized by mutations in the p53 gene. © 2001 Cancer Research Campaign http://www.bjcancer.com


*British Journal of Cancer* (2001) **84**(8), 1070–1075
© 2001 Cancer Research Campaign
doi: 10.1054/ bjoc.2001.1728, available online at http://www.idealibrary.com on IDEAL®

http://www.bjcancer.com

### Hypoxia in human soft tissue sarcomas: Adverse impact on survival and no association with *p53* mutations

**M Nordsmark[1,2], J Alsner[1], J Keller[3], OS Nielsen[2], OM Jensen[4], MR Horsman[1] and J Overgaard[1]**

[1]Danish Cancer Society Department of Experimental Clinical Oncology and Departments of [2]Oncology, [3]Orthopaedic Surgery and [4]Pathology, Aarhus University Hospital, Denmark

**Summary** Clinical and experimental studies have suggested that tumour hypoxia is associated with poor treatment outcome and that loss of apoptotic potential may play a role in malignant progression of neoplastic cells. The tumour suppressor gene *p53* induces apoptosis under certain conditions and microenvironmental tumour hypoxia may select for mutant tumour cells with diminished apoptotic potential due to lack of p53 function. The aim of this study was to evaluate the prognostic relevance of oxygenation status for treatment outcome and to compare pre-treatment tumour oxygenation measurements were done in 31 of those by PCR using DNA extracted from paraffin-embaedded sections ($n$ = 2) or frozen biopsies ($n$ = 29). The overall median of the tumour median $pO_2$ was 19 mmHg (range 1–58 mmHg). Only 6 tumours had functional p53 mutations and no association was found between mutant p53 and tumour hypoxia. Five out of 6 STS with lower histopathological grade were well-oxygenated whereas high-grade STS were both hypoxic and well-oxygenated. At a median follow-up of 74 months, 16 patients were still alive among 28 available for survival analysis. When stratifying into hypoxic and well-oxygenated tumours patients with the most hypoxic tumours has a statistically poorer disease-specific and overall survival at 5 years. In conclusion hypoxia was an indicator for both a poorer disease specific and overall survival in human STS but hypoxic tumours were not characterized by mutations in the p53 gene. © 2001 Cancer Research Campaign http://www.bjcancer.com

**Keywords**: soft tissue sarcoma; *p53*; hypoxia; polarographic oxygen electrode; tumour oxygenation; predictive assay; survival

It is well known that hypoxia is a common feature of solid tumours and that neoplastic cells can survive under conditions of low oxygenation as well as glucose deprivation (Vaupel et al, 1991). Clinical and experimental studies have demonstrated that tumour hypoxia causes resistance to radiation therapy, some chemotherapy agents, and also to surgery alone (Moulder and Rockwell, 1984; Durand, 1991; Höckel et al, 1996; Nordsmark et al, 1996a). Recently, low apoptotic potential was demonstrated in hypoxic highly aggressive human tumours (Höckel et al, 1999). These clinical results suggested that hypoxia could induce malignant progression, but is hypoxia really a driving force in neoplastic transformation or is it simply a consequence of rapid tumour cell proliferation accompanied with insufficient blood supply? In-vitro studies have provided interesting results and shown that both normal and cancer cells alter gene expression under hypoxic conditions (Sutherland, 1996, Dachs and Tozer, 2000), supporting the role of hypoxia in malignant progression.

The tumour suppressor gene *p53* induces apoptosis under certain conditions and tumour hypoxia was found to select for p53 mutant tumour cells with diminished apoptotic potential (Graeber et al, 1996). These findings led us to determine the prognostic value of pre-treatment tumour oxygenation measurements on survival after long-term follow-up and to investigate both tumour oxygenation and p53 status in untreated human soft tissue sarcomas in order to test whether hypoxic tumours might be apoptotic incompetent due to mutations in the *p53* gene.

*Received 8 May 2000*
*Revised 16 January 2001*
*Accepted 25 January 2001*

*Correspondence to:* M Nordsmark

### MATERIALS AND METHODS

### Patients

The study involved 33 patients with primary extra-visceral soft tissue sarcomas, referred to the Bone and Soft Tissue Sarcoma Centre, Aarhus University Hospital, Denmark from February 1992 to May 1999. Using 3 eligibility criteria: (1) age ≥ 18 years, (2) tumour accessible for invasive oxygen electrode measurements and (3) minimum tumour diameter of 2 cm, most of the 33 patients matched a background population of 316 primary STS referred to our centre between January 1979 and July 1993 (Vraa et al, 1998). The local ethical committee gave approval and all patients gave informed consent according to the Helsinki Declaration II. Median age at the time of diagnosis was 54 years (range 23–87 years). Sixteen were female, 12 were tobacco users, 18 did not use tobacco and in 3 cases smoking history was not available. Two patients had recurrent STS. Tumour sites were extremities ($n$ = 26), back ($n$ = 5) and abdomen and thorax ($n$ = 2).

Based on examinations of biopsy and/or surgical specimens the group pathologist determined the histopathological diagnosis and tumour grade as previously described (Jensen et al, 1991).

### Tumour oxygenation measurements

Tumour oxygen partial pressure ($pO_2$) was measured prior to treatment using computerized polarographic oxygen-sensitive electrodes (Eppendorf, Germany). This method has previously been described in detail and found reliable and feasible in a whole range of human tumours (Vaupel et al, 1991; Nordsmark et al, 1994). Routine magnetic resonance imaging (MRI) provided anatomical details about the tumour localization, volume and depth below the skin surface and this information was used during the $pO_2$ measurement procedure to ensure that the length of each needle electrode track was restricted to tumour tissue. Based on results from a previous variance analysis a minimum of 3 electrode tracks were required in order to reduce intra-tumour variability as compared to tumour-to-tumour variability (Nordsmark et al, 1994). Among the 33 STS the median number of $pO_2$ values was 184 (range 64–381). These measurements were sampled along a median of 5 electrode tracks (range 3–7) per tumour. Tumour oxygenation status was evaluated by the median tumour $pO_2$. From venous blood samples the total haemoglobin level was measured using a blood gas analyser (Radiometer OSM3, Denmark).

Some of the patients with Eppendorf data reported here have previously been published as a comparison between tumour oxygenation status and cell kinetic parameters involving 21 STS (Nordsmark et al, 1996b) and as a feasibility study of tumour oxygenation assessment and $^{31}$PMRS involving 25 STS (Nordsmark et al, 1997).

### p53 mutation analysis

Biopsy material was available for p53 mutation analysis in 31 STS. In the remaining 2 cases only paraffin-embedded tissue was available but it was not possible to extract enough tissue for analysis. DNA was successfully extracted from paraffin-embedded sections (*n* = 2) (QIAamp DNA Mini Kit, Qiagen, Valencia, CA) or frozen biopsies (*n* = 29) (Puragene DNA Isolation Kit, Gentra Systems, Minneapolis, MN). The entire coding region and all exon/intron boundaries of p53 were analysed by denaturing gradient gel electrophoresis (DGGE). Twelve sets of primers for amplification and DGGE analysis of exons 2–11 (including overlapping amplicons for exons 4 and 5) have been described previously (Guldberg et al, 1997). Amplification reactions were carried out by 38 rounds of thermal cycling (94˚C for 20 s, 62˚C for 20 s, and 72˚C for 20 s) in final volumes of 15 µl containing 10 mM Tris/HCl (pH 8.3), 50 mM KCl, 1.5 mM $MgCl_2$, 0.02% gelatine, 0.2 mM cresol red, 12% sucrose, 5% DMSO, 100 µM of each dNTP, 0.6 µM of each primer, 100 ng DNA, and 0.5 U AmpliTaq DNA polymerase (Applied Biosystems, Foster City, CA). For DGGE analysis, PCR products were run on 6% polyacrylamide gels containing various gradients of urea and formamide (100% denaturant consisted of 7 M urea and 40% formamide). As a modification to a previous approach (Guldberg et al, 1997), the gradient ranges were narrowed and adapted to each exon to increase the separation between mutant and wild-type alleles. The gradient ranges were for exon 5: 40–80%, for exon 6: 25–65%, for exons 7 and 8: 35–75%, and for exon 9: 20–60%, respectively. The gels were run for 1050 V hours in 1 × TAE buffer at a constant temperature of 58˚C. Following electrophoresis, gels were stained in 1 × TAE buffer containing 2 µg per ml ethidium bromide and analysed under UV-transillumination. Mutant heteroduplex or homoduplex bands were excised and re-amplified as described (Guldberg et al, 1997). Sequencing of PCR products was performed with the BigDye DyeTerminator Cycle Sequencing Kit and analysed on an ABI 310 (Applied Biosystems, Foster City, CA).

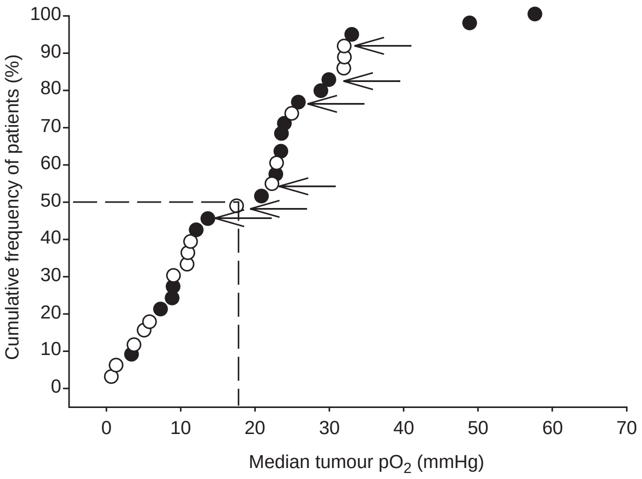

**Figure 1** Median tumour $pO_2$ from 33 patients plotted on a cumulative frequency scale. Open symbols (○) indicate patients who have died and filled symbols (●) patients alive. Arrows indicate the 6 tumours with p53 mutations

### Treatment and follow up

Among the 33 patients the 2 STS with recurrent disease were not included in the survival analysis. Of the remaining 31 patients with primary STS 3 were not evaluated due to incomplete treatment (*n* = 1), and breast cancer (*n* = 2). This leaves 28 cases for survival analysis of tumour oxygenation as a prognostic marker. Twelve patients were treated by surgery alone using wide margins and limb conservation as previously described (Vraa et al, 1998). Adjuvant radiation therapy (RT) was given as preoperative RT to 11 patients and as postoperative RT in 5 cases. The radiation doses were 50 Gy in 25 fractions (*n* = 15) and 60 Gy in 30 fractions (*n* = 1).

The initial follow-up included physical examination and chest X-ray 2 months after the end of treatment and subsequent controls were done regularly until 5 years. In individual cases consultation was done more often than routinely scheduled. Local recurrence or distant metastases were fully evaluated and if possible patients with relapse were offered either surgery or chemotherapy. The treatment endpoints were overall survival and disease-specific survival defined as death following treatment, death due to STS, or death from other diseases with tumour or metastasis. Tumour control was defined as complete and persistent regression and no sign of disseminated disease. Follow-up was evaluated from the first day of treatment until December 1, 1999 or until death. The median follow-up for all patients was 74 months (range 13–83) with a minimum follow-up of 13 months among the survivors.

### Statistical analysis

Disease-specific and overall 5-year actuarial survival was evaluated by Kaplan–Meier plots and differences were estimated by the log-rank analysis using the BMDP statistical program. The Cox proportional hazard model was used for univariate analysis of median tumour $pO_2$ as a prognostic parameter. Comparisons between patient and tumour characteristics among survivors and the dead, and among patients with hypoxic versus well-oxygenated tumours was done by the Wilcoxon signed rank test or Chi-square test. In all cases a 2-sided significance level of 5% was used.

© 2001 Cancer Research Campaign

**Table 1** Characterization of p53 mutations

| Pt | Mutation | Exon | Structural change | Presumed functional change |
|---|---|---|---|---|
| 1 | g.13429-13469del[a] | 6 | Splice deficiency | Splice deficiency, loss of function |
| 2 | c.880G>T[b] | 8 | E294X | Nonsense mutation, loss of function |
| 3 | c.659A>G | 6 | Y220C | Missense mutation in the short loop between the S7 and S8 strand,[c] loss of function (transactivation activity[d]) |
| 4 | c.469G>T | 5 | V157F | Missense mutation in the S4 strand[c], loss of function (transactivation activity[e]) |
| 5 | c.625-626del | 6 | R209delX214 | Frameshift mutation, loss of function |
| 6 | c.844C>G | 8 | R282G | Missense mutation in the H2 helix of the LSH motif[c], loss of function[c] |

[a]g.denotes genomic number (AC no. U94788). [b]c. denotes cDNA number (AC no M14694). [c]According to Cho et al (1994). [d]According to Di Como and Prives (1998). [e]According to Obata et al (2000).

**Table 2** Survival status and patient and tumour characteristics

| | All median (range) | Alive median (range) | Dead median (range) | *P* value |
|---|---|---|---|---|
| No of patients | 28 | 16 | 12 | |
| Age (years) | 59 (30–87) | 50 (33–80) | 67 (30–87) | NS |
| Sex (male/female) | 15/13 | 8/8 | 7/5 | NS |
| Hb total (mmol $l^{-1}$) | 8.6 (5.5–9.7) | 8.7 (7.4–9.7) | 8.4 (5.5–9.6) | NS |
| Median tumour $pO_2$ (mmHg) | 19 (1–58) | 24 (3–58) | 10 (1–32) | NS* |
| Tumour size (cm) | 10 (3–20) | 9 (3–20) | 10 (4–20) | NS |
| Functional p53 mutation | 6 | 3 | 3 | |
| Histopathology | | | | |
| Grade I–II | 6 | 6 | 0 | |
| Grade III | 22 | 10 | 12 | |
| Treatment | | | | |
| Surgery | 12 | 5 | 7 | |
| Adjuvant RT | 16 | 11 | 5 | |

NS = not significant; *$P$ = 0.08.

**Table 3** Oxygenation status and patient and tumour characteristics

| | All median (range) | Well-oxygenated median (range) | Hypoxic median (range) | *P* value |
|---|---|---|---|---|
| No of patients | 28 | 14 | 14 | |
| Age (years) | 59 (30–87) | 52 (33–87) | 64 (30–81) | NS |
| Sex (male/female) | 15/13 | 5/9 | 10/4 | NS |
| Hb total (mmol $l^{-1}$) | 8.6 (5.5–9.7) | 8.7 (7.6–9.4) | 8.2 (5.5–9.7) | NS |
| Median tumour $pO_2$ (mmHg) | 19 (1–58) | 30 (21–58) | 8 (1–18) | <0.01 |
| Tumour size (cm) | 10 (3–20) | 8 (3–20) | 10 (4–20) | NS |
| Functional p53 mutation | 6 | 4 | 2 | |
| Histopathology | | | | |
| Grade I–II | 6 | 5 | 1 | |
| Grade III | 22 | 9 | 13 | |
| Treatment | | | | |
| Surgery | 12 | 5 | 7 | |
| Adjuvant RT | 16 | 9 | 7 | |

NS = not significant.

### RESULTS

Wild-type p53 was found in 25 tumours and mutations in 6 cases with primary STS. The presumed functional change in each mutation is listed in Table 1. Figure 1 shows the median tumour $pO_2$ of 33 individual tumours plotted on a frequency scale to illustrate the tumour-to-tumour variability and the relationship to p53 status and survival. No association was found between the median tumour $pO_2$ and p53 status. Tumour oxygenation status varied between all 33 soft tissue sarcomas with an overall median = 19 mmHg (range 1–58

© 2001 Cancer Research Campaign

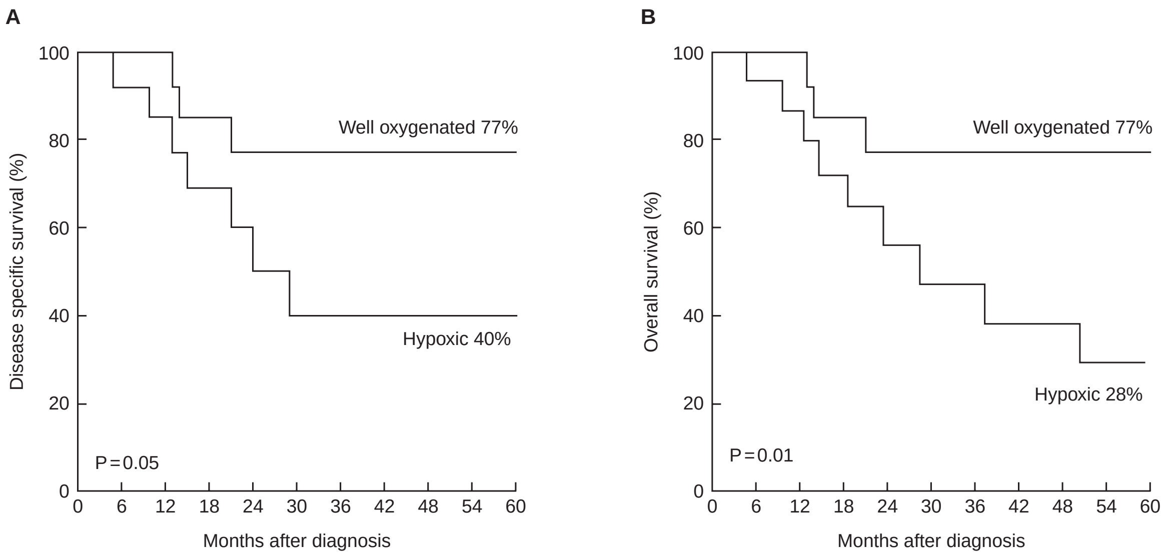

**Figure 2** Tumour oxygenation was evaluated by the overall median tumour $pO_2$. Tumours with median $pO_2$ above 19 mmHg were classified as well-oxygenated and below or equal to 19 mmHg as hypoxic. 5 year disease-specific and overall survival rates were estimated by actuarial tumour control probability using Kaplan–Maier estimates. Patients with the most hypoxic tumours had a statistically significant poorer disease specific survival probability (Figure 2A; $P = 0.05$) and poorer overall survival probability (Figure 2 B; $P = 0.01$) when compared to well oxygenated tumours.

mmHg).

At the time of analysis 16 patients were still alive. Ten died from STS with pulmonary metastasis, 1 from stroke and 1 from cardiac disease. Among the survivors 10 patients completed the planned 5 year period of follow-up and none of these had any sign of recurrence. One patient with distant metastasis is still alive 4 years after time of relapse. 12 patients died with an overall median $pO_2$ of 10 mmHg (range 1–32 mmHg) as compared to 24 mmHg (range 3–58 mmHg) among the 16 survivors (Wilcoxon signed rank test, $P = 0.12$). The groups of survivors and non-survivors were equally distributed with respect to age, sex, tumour size, treatment strategy, haemoglobin and smoking history, whereas histological grade I and II tumours were more frequent among survivors. Six among 28 (19%) expressed mutant p53 and 3 (10%) of these died, whereas 22 among 28 (79%) of patients had wild-type p53 and 11 (39%) of these died. Some of these parameters are listed in Table 2. Identical tumour characteristics were compared according to oxygenation status (Table 3). The overall median among the 28 cases amenable for survival analysis was 19 mmHg. Tumours above a median $pO_2$ of 19 mmHg were classified as well-oxygenated and ≤19 mmHg as hypoxic. It was seen that well-oxygenated tumours were slightly more common among females. Interestingly, all 6 patients with grade I–II tumours were alive by the time of analysis and only 1 of these tumours was hypoxic (median $pO_2$ ≤19 mmHg), whereas grade III tumours ($n = 22$) represented both hypoxic ($n = 13$) and well-oxygenated ($n = 9$) tumours. Tumour grade reached statistical significance as a prognostic variable in a univariate analysis with overall survival as the endpoint ($P = 0.03$). Also, the median tumour $pO_2$ was a significant prognostic factor ($P = 0.01$) in a univariate analysis with a decrease in median $pO_2$ related to a decrease in overall survival probability. Using a Kaplan–Meier analysis patients were stratified into well oxygenated or hypoxic based on overall median $pO_2$ (Figure 2). The hypoxic group had a significantly poorer disease-specific survival of 40% compared to 77% for well-oxygenated tumours ($P = 0.05$). Likewise patients with hypoxic tumours had a significantly poorer overall survival probability of 28% versus 77% ($P = 0.01$) at 5 years.

### DISCUSSION

The current study showed that hypoxic primary STS had a poorer overall survival rate after 5 years of follow-up. Patients received pre- or post-operative RT or surgery alone and were followed for a median of 6 years. This result is in agreement with a previous report in primary STS by Brizel et al where the follow up was relatively short (median 1 year) and where the endpoint was freedom from metastases (Brizel et al, 1996). Similar results were obtained from a range of studies in uterine cervix carcinomas and squamous cell carcinoma of the head and neck (Hockel et al, 1996; Nordsmark et al, 1996a; Fyles et al, 1998; Adam et al, 1999; Brizel et al, 1999; Knocke et al, 1999; Stadler et al, 1999; Nordsmark and Overgaard, 2000; Rudat et al, 2000; Sundfor et al, 2000).

It was suggested that hypoxia could induce malignant progression in human cervix carcinoma (Höckel et al, 1996) and soft tissue sarcoma (Brizel et al, 1996). But what is the clinical and experimental evidence that hypoxia induces malignant progression? Certainly, there is increasing evidence that treatment failure among patients with hypoxic tumours is not an effect of radiation-resistant hypoxic cells alone but whether this is related to malignant progression still remains to be proven. The experimental in-vitro data are encouraging. An increasing number of studies have reported that different genes are up- or down-regulated during hypoxia and fluctuating oxygenation (for review Dachs and Tozer, 2000). Interestingly, melanoma cells in-vitro progressed from a benign to a highly malignant phenotype after exposure to sequential rounds of hypoxia (Stackpole et al, 1994).

The clinical data available are limited in number of patients and provide more indirect evidence that hypoxia may induce metastases. We previously found an inverse relationship between the tumour cell potential doubling time (Tpot) and the median tumour

© 2001 Cancer Research Campaign

$pO_2$ in human soft tissue sarcomas and we hypothesized that high proliferation rate was confined to more hypoxic tumours (Nordsmark et al, 1996b). In hypoxic human cervix carcinomas low apoptotic index was associated with highly aggressive tumours (Höckel et al, 1999). Hypoxia inducible factor 1 (HIF-1α) was up-regulated in a range of human tumours (Zohng et al, 1999) and it was shown that over-expression of HIF-α was a marker for poor prognosis in early stage uterine cervix carcinoma (Birner et al, 2000). HIF-1α is an $O_2$-regulated subunit of a more complex protein that can be assessed by immunohistochemistry.

Experimental studies have suggested that apoptotic cell kill was compromised by hypoxia in mutant p53 cells. Graeber et al (1996) showed that hypoxia-induced apoptosis in oncogenically transformed cells and loss of the *p53* suppressor gene made cells less susceptible to hypoxia-induced cell death. They also demonstrated that hypoxia co-localized with apoptosis in-vivo (Graeber et al, 1996). We tested this hypothesis in human STS but found no association between mutant p53 and hypoxia, which was in agreement with the results obtained by Birner et al (2000). These results can of course reflect the biological true situation but other factors may play a role. The current study was small in terms of the number of patients and therefore the statistical power was poor, hence our results are to be considered as hypothesis-generating. The patient number in the Birner study was higher but a weakness of that study was that they measured p53 protein by immunohistochemistry and using that technique certain forms of mutant p53 can be missed. There is general agreement that mutant p53 is an indicator for poor prognosis in some human cancers (Klijn, 1997), but it is also well known that cell control can be interrupted in cases where wild-type p53 may not be functional due to mutations in other genes (Vogelstein et al, 2000).

Histopathological grade is a classical prognostic indicator for survival in STS (Jensen et al, 1991; Vraa et al, 1998). We found that all 6 patients with grade I or II tumours were alive and 5 of those had less hypoxic tumours. Histopathological grade is in general poorly reported in studies that have evaluated the prognostic value of oxygenation status. One study in breast cancer reported that 6 out of 7 cases with lower-grade tumours were well oxygenated with a median tumour $pO_2$ above 22 mmHg (Hohenberger et al, 1998). Two studies in squamous cell carcinomas of either uterine cervix or head and neck found no correlation between hypoxia and histological grade (Höckel et al, 1996; Nordsmark et al, 1996a). Even if multivariate analyses were performed none of the trials had sufficient statistical power to test the prognostic value of tumour hypoxia and histological grade on survival. In addition, the current study did not show that hypoxia predisposes to metastases independent of grade.

In conclusion, hypoxia was an indicator for poor prognosis in soft tissue sarcomas as patients with the most hypoxic tumours were associated with a poorer disease-specific and overall survival probability. Lower-grade STS were in general well-oxygenated. Finally, hypoxic tumours were not characterized by mutations in the *p53* gene.

### ACKNOWLEDGEMENTS

This work was supported by the Danish Cancer Society, The Clinical Research Unit at the Oncology Centre, Aarhus University Hospital and Mrs Agnes Niebuhr Anderssons Cancer Research Foundation. The authors are thankful to Ms A Baden, Ms K Hillebrandt, Ms I M Johansen, Mr MJ Johansen, Ms B Kierkegaard and Ms IM Thuesen for excellent technical assistance.

### REFERENCES

Adam MF, Gabalski EC, Bloch DA, Oehlert JW, Brown JM, Elsaid AA, Pinto HA and Terris DJ (1999) Tissue oxygen distribution in head and neck cancer patients. *Head Neck* **21**: 146–153

Birner P, Schindl M, Obermair A, Plank C, Breitenecker G and Oberhuber G (2000) Overexpression of hypoxia-inducible Factor 1α is a marker for an unfavorable prognosis in early-stage invasive cervical cancer. *Cancer Res* **60**: 4693–4696

Brizel DM, Scully SP, Harrelson JM and Dewhirst MW (1996) Tumor oxygenation predicts for the likelihood of distant metastasis in human soft tissue sarcoma. *Cancer Res* **56**: 941–943

Brizel DM, Dodge RK, Clough RW and Dewhirst MW (1999) Oxygenation of head and neck cancer: changes during radiotherapy and impact on treatment outcome. *Radiother Oncol* **53**: 113–117

Cho Y, Gorina S, Jeffrey PD and Pavletich NP (1994) Crystal structure of a p53 tumour suppressor-DNA complex: understanding tumorigenic mutations. *Science* **265**: 346–355

Dachs GU and Tozer GM (2000) Hypoxia modulated gene expression: angiogenesis, metastasis and therapeutic exploitation *Eur J Cancer* **36**: 1649–1660

Di Como CJ and Prives C (1998) Human tumor-derived p53 proteins exhibit binding site selectivity and temperature sensitivity for transactivation in a yeast-based assay. *Oncogene* **16**: 2527–2539

Durand RE (1991) Keynote address: the influence of microenvironmental factors on the activity of radiation and drugs. *Int J Radiat Oncol Biol Phys* **20**: 253–258

Fyles AW, Milosevic M, Wong R, Kavanagh MC, Pintilie M, Sun A, Chapman W, Levin W, Manchul L, Keane TJ and Hill RP (1998) Oxygenation predicts radiation response and survival in patients with cervix cancer. [published erratum appears in Radiother Oncol 1999 Mar; 50(3):371]. *Radiother Oncol* **48**: 149–156

Graeber TG, Osmanian C, Jacks T, Housman DE, Koch CJ, Lowe SW and Giaccia AJ (1996) Hypoxia-mediated selection of cells with diminished apoptotic potential in solid tumours. *Nature* **379**: 88–91

Guldberg P, Nedergaard T, Nielsen HJ, Olsen AC, Ahrenkiel V and Zeuthen J (1997) Single-step DGGE-based mutation scanning of the p53 gene: application to genetic diagnosis of colorectal cancer. *Hum Mutat* **9**: 348–355

Hockel M, Schlenger K, Aral B, Mitze M, Schaffer U and Vaupel P (1996) Association between tumor hypoxia and malignant progression in advanced cancer of the uterine cervix. *Cancer Res* **56**: 4509–4515

Hockel M, Schlenger K, Hockel S and Vaupel P (1999) Hypoxic cervical cancers with low apoptotic index are highly aggressive. *Cancer Res* **59**: 4525–4528

Hohenberger P, Felgner C, Haensch W and Schlag PM (1998) Tumor oxygenation correlates with molecular growth determinants in breast cancer. *Breast Cancer Res Treat* **48**: 97–106

Jensen OM, Hogh J, Ostgaard SE, Nordentoft AM and Sneppen O (1991) Histopathological grading of soft tissue tumours. Prognostic significance in a prospective study of 278 consecutive cases. *J Pathol* **163**: 19–24

Klijn JGM (1997) *Prognostic and predictive value of p53*. European school of Oncology, Scientific Updates 1. Elsevier Science BV: Amsterdam

Knocke TH, Weitmann HD, Feldmann HJ, Selzer E and Potter R (1999) Intratumoral pO2-measurements as predictive assay in the treatment of carcinoma of the uterine cervix. *Radiother Oncol* **53**: 99–104

Moulder JE and Rockwell S (1984). Hypoxic fractions of solid tumours: experimental techniques, methods of analysis, and a survey of excisting data. *Int J Radiat Oncol Phys* **10**: 695–712

Nordsmark M and Overgaard J (2000) A confirmatory prognostic study on oxygenation status and loco-regional control in advanced head and neck squamous cell carcinoma treated by radiation therapy. *Radiother Oncol* **57**: 39–43

Nordsmark M, Bentzen SM and Overgaard J (1994) Measurement of human tumour oxygenation status by a polarographic needle electrode. An analysis of inter- and intratumour heterogeneity. *Acta Oncol* **33**: 383–389

Nordsmark M, Overgaard M and Overgaard J (1996a) Pretreatment oxygenation predicts radiation response in advanced squamous cell carcinoma of the head and neck. *Radiotherapy and Oncology* **41**: 31–39

Nordsmark M, Höyer M, Keller J, Nielsen OS, Jensen OM and Overgaard J (1996b) The relationship between tumour oxygenation and cell proliferation in human soft tissue sarcomas. *Int J Radiat Oncol Biol Phys* **35**: 701–708

Nordsmark M, Keller J, Nielsen OS, Lundorf E and Overgaard J (1997) Tumour

 *© 2001 Cancer Research Campaign*

oxygenation assessed by polarographic needle electrodes and bioenergetic status measured by 31P magnetic resonance spectroscopy in human soft tissue tumours. *Acta Oncol* **36**: 565–571

Rudat V, Vanselow B, Wollensack P, Bettscheider C, Osman-Ahmet S, Eble MJ and Dietz A (2000) Repeatability and prognostic impact of the pretreatment pO2 histography in patients with advanced head and neck cancer. *Radiother Oncol* **57**: 31–37

Stackpole CW, Groszek L and Kalbag SS (1994) Benign-to-malignant B 16 melanoma progression induced in two stages in vitro by exposure to hypoxia. *J Natl Cancer Inst* **86**: 361–367

Stadler P, Becker A, Feldmann HJ, Hansgen G, Dunst J, Wurschmidt F and Molls M (1999) Influence of the hypoxic subvolume on the survival of patients with head and neck cancer. *Int J Radiat Oncol Biol Phys* **44**: 749–754

Sundfor K, Lyng H, Trope CG and Rofstad EK (2000) Treatment outcome in advanced squamous cell carcinoma of the uterine cervix: relationships to pretreatment tumor oxygenation and vascularization. *Radiother Oncol* **54**: 101–107

Sutherland RM, Ausserer WA, Murphy BJ and Laderoute KR (1996) Tumor Hypoxia and heterogeneity: Challenges and opportunities for the future. *Sem Rad Oncol* **6**: 59–70

Vaupel P, Schlenger K, Knoop C and Höckel M (1991) Oxygenation of human tumors: evaluation of tissue oxygen distribution in breast cancers by computerized O2 tension measurements. *Cancer Res* **51**: 3316–3322

Vogelstein B, Lane D and Levine AJ (2000) Surfing the p53 network. *Nature* **408**: 307–310

Vraa S, Keller J, Nielsen OS, Sneppen O, Jurik AG and Jensen OM (1998) Prognostic factors in soft tissue sarcomas: the Aarhus experience. *Eur J Cancer* **34**: 1876–1882

© 2001 Cancer Research Campaign
